# *BolTLP1*, a Thaumatin-like Protein Gene, Confers Tolerance to Salt and Drought Stresses in Broccoli (*Brassica oleracea* L. var. *Italica*)

**DOI:** 10.3390/ijms222011132

**Published:** 2021-10-15

**Authors:** Lixia He, Lihong Li, Yinxia Zhu, Yu Pan, Xiuwen Zhang, Xue Han, Muzi Li, Chengbin Chen, Hui Li, Chunguo Wang

**Affiliations:** 1 Department of Genetics and Cell Biology, College of Life Sciences, Nankai University, Tianjin 300071, China; helx@mail.nankai.edu.cn (L.H.); llh348536673@126.com (L.L.); 18322712771@163.com (Y.Z.); Pan1997666@126.com (Y.P.); zxw20150316@163.com (X.Z.); hx18322106151@126.com (X.H.); chencb@nankai.edu.cn (C.C.); 2 College of Horticulture and Landscape, Tianjin Agricultural University, Tianjin 300384, China; mz5872991001@163.com; 3 State Key Laboratory of Tree Genetics and Breeding, Northeast Forestry University, Harbin 150040, China; 4 State Key Laboratory of Vegetable Germplasm Innovation, Tianjin Academy of Agricultural Sciences, Tianjin 300381, China

**Keywords:** thaumatin-like proteins (TLPs), bolTLP1, broccoli, salt stress, drought stress

## Abstract

Plant thaumatin-like proteins (TLPs) play pleiotropic roles in defending against biotic and abiotic stresses. However, the functions of TLPs in broccoli, which is one of the major vegetables among the *B. oleracea* varieties, remain largely unknown. In the present study, *bolTLP1* was identified in broccoli, and displayed remarkably inducible expression patterns by abiotic stress. The ectopic overexpression of *bolTLP1* conferred increased tolerance to high salt and drought conditions in *Arabidopsis*. Similarly, *bolTLP1*-overexpressing broccoli transgenic lines significantly improved tolerance to salt and drought stresses. These results demonstrated that *bolTLP1* positively regulates drought and salt tolerance. Transcriptome data displayed that *bolTLP1* may function by regulating phytohormone (ABA, ethylene and auxin)-mediated signaling pathways, hydrolase and oxidoreductase activity, sulfur compound synthesis, and the differential expression of histone variants. Further studies confirmed that RESPONSE TO DESICCATION 2 (RD2), RESPONSIVE TO DEHYDRATION 22 (RD22), VASCULAR PLANT ONE-ZINC FINGER 2 (VOZ2), SM-LIKE 1B (LSM1B) and MALATE DEHYDROGENASE (MDH) physically interacted with bolTLP1, which implied that bolTLP1 could directly interact with these proteins to confer abiotic stress tolerance in broccoli. These findings provide new insights into the function and regulation of bolTLP1, and suggest potential applications for bolTLP1 in breeding broccoli and other crops with increased tolerance to salt and drought stresses.

## 1. Introduction

Thaumatin-like proteins (TLPs) are widely distributed in plants, animals and fungi [1], and are named for their high sequence similarity to the thaumatin protein initially identified in shrub *Thaumatococcus daniellii* Benth [2]. Most TLPs contain about 200 amino acids and have a molecular mass ranging from 21 to 26 kDa, except the poplar TLPs, most of which are 24 to 34 kDa in size [1,3,4]. In plants, TLPs are classified as pathogenesis-related protein family 5 (PR-5), one out of the 17 defense-related PR protein families [5,6]. Examination of genome databases revealed at least 37 *TLPs* in *Oryza sativa*, 33 in *Zea mays*, 18 in *Vitis vinifera*, 25 in *Arabidopsis thaliana*, 28 in *Prunus persica* and 42 in *Populus trichocarpa* [4,7]. However, the functions of the majority of TLPs are still unknown. Previous reports demonstrated that a few plant TLPs play important roles in defending against pathogen infection, particularly fungal infection. A 20 kDa TLP from the French bean legumes displays considerable resistance against *Fusarium oxysporum*, *Pleurotus ostreatus* and *Coprinus comatus* [8]. In *Cassia didymobotrya*, a 23 kDa TLP was identified, which exerts antifungal activity toward some Candida species [9]. *TaLr35PR5*, a *TLP* from wheat, was involved in *Lr35*-mediated adult wheat defense in response to leaf rust attack [10]. The recombinant expression of *Mald2*, a *TLP* identified in apple, conferred antifungal activity against *Fusarium oxysporum* and *Penicillium expansum* in *Nicotiana benthamiana* [11]. In tobacco, the ectopic overexpression of *PpTLP* from *Pyrus pyrifolia* or *AdTLP* from *Arachis diogoi* can enhance resistance to *Rhizoctonia solani* [12,13]. Transgenic canola, wheat and banana overexpressing *TLPs* from rice all exhibit increased resistance to diverse pathogens [14,15,16]. In addition to resistance against biotic stress, some *TLPs* in plants also respond to abiotic stress. Transgenic tobacco plants ectopically expressing *AdTLP* not only have enhanced resistance to *Rhizoctonia solani*, but also have improved tolerance to salt and oxidative stresses [13]. The ectopic expression of *ObTLP1* isolated from *Ocimum basilicum* enhances tolerance to dehydration and salt stresses in *Arabidopsis* [17]. Under drought stress, *TLPs* showing inducible expression patterns were also identified in carrot and tea, suggesting that these *TLPs* could be involved in drought response [18,19]. In addition, *TLPs* have also been demonstrated to play roles in regulating plant growth and development including fruit maturation, lentinan degradation and seed development [20,21,22,23,24]. These investigations indicate that *TLPs* have undergone functional diversification in plant species, and play pleiotropic roles in response to biotic and abiotic stresses as well as in the regulation of growth and development [4,7].

Broccoli (*Brassica oleracea* L. var. *italica*) is one of the major vegetables among the *brassica oleracea* varieties, and is widely planted in Asia, Europe and North America [25]. Soil salinization and drought are two potential threats in planting broccoli, which can result in curd yield reduction or even no harvest [26]. Breeding broccoli varieties with high tolerance to drought and salt is an important target for broccoli breeders. Several studies related to the responses to these two abiotic stresses were reported in broccoli and other species of *Brassica*. Broccoli pre-treating with SA and chitosan showed the highest drought stress recovery in a dose-dependent manner [27]. Drought-tolerant broccoli cultivars presented higher levels of methionine and ABA than that of the drought-sensitive cultivars [28]. 42 putative known and 39 putative candidate miRNAs displayed differentially expressed patterns between control and salt-stressed broccoli [29]. *Bra-botrytis-ERF056* from cauliflower was demonstrated to increase tolerance to salt and drought stresses in overexpressing *bra-botrytis-ERF056 Arabidopsis* [30]. *BrEXLB1* is involved in drought tolerance in *Brassica rapa* [31]. *BnaABF2* from *Brassica napus* confers enhanced drought and salt tolerance in transgenic *Arabidopsis* [32]. *BolOST1* was dramatically induced by drought and high-salt stress, and the ectopic expression of *BolOST1* restored the drought-sensitive phenotype of *ost1* [33]. However, natural response to salt and drought stresses in broccoli is still largely unknown. In our previous transcriptome data analysis, a *TLP* gene named *bolTLP1* was identified in broccoli which exhibited a significant positive response to salt stress. However, the role of *bolTLP1* is unknown. In the present study, the full-length cDNA of *bolTLP1* was cloned, and its expression patterns under salt stress and drought stress were analyzed. Transgenic *Arabidopsis* and broccoli overexpressing *bolTLP1* were generated. The phenotypes of these *bolTLP1* overexpressing transgenic plants under salt and drought stresses were observed. The genes and regulatory processes involved in *bolTLP1*-mediated tolerance to abiotic stress were explored.

## 2. Results

### 2.1. BolTLP1 Was Identified in Broccoli

**Figure 1:**
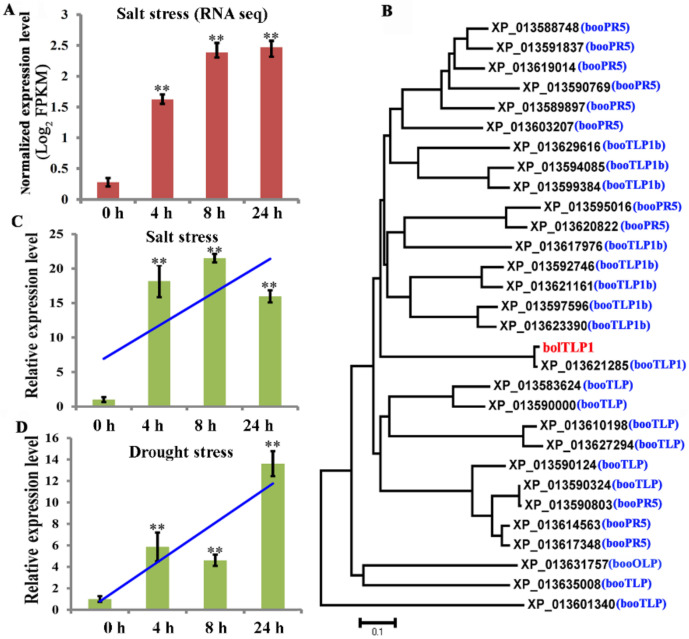
Phylogenetic tree and expression profiles of *bolTLP1* under salt and drought stresses. (**A**): Expression profiles of *bolTLP1* at 0 h (control), 4 h, 8 h and 24 h after salt stress (200 mM NaCl treatment) detected by RNA-seq. (**B**): Phylogenetic analysis of *bolTLP1* and genes with a TLP domain from *Brassica oleracea* var. *oleracea* (Bootstrap value = 1000). The bar indicated the genetic distance of 0.1. The red font showed the position of bolTLP1. (**C**): Expression profiles of *bolTLP1* at 0 h (control), 4 h, 8 h and 24 h after salt stress (200 mM NaCl treatment) detected by qRT-PCR. (**D**): Expression profiles of *bolTLP1* at 0 h (control), 4 h, 8 h and 24 h after mimetic drought stress (300 mM mannitol treatment). The blue lines indicated the expression trends of *bolTLP1* under salt and drought stresses. ** indicated the significantly differential expression levels of *bolTLP1* under salt and drought stresses (*p* < 0.01).

In previous comparative transcriptome sequence analysis to identify genes involved in salt stress response in broccoli, *bolTLP1* was found to be rapidly induced under salt stress condition (Figure 1A). In the present study, the full-length cDNA of *bolTLP1* was identified based on the ESTs of this gene from broccoli transcriptome data. Sequence analysis indicated that the coding region of *bolTLP1*comprises 696 bp and encodes a predicted 231-amino acid protein, which contained a conserved TLP domain. The phylogenetic analysis indicated that *bolTLP1* and 1 out of the 29 *TLP* genes identified in *Brassica oleracea* var. *oleracea* (XP_013621285), are classified into a separate group, and they appear to show far genetic distance with other *TLP* genes (Figure 1B).

### 2.2. BolTLP1 Positively Responded to Salt and Drought Stresses in Broccoli

The expression pattern of *bolTLP1* under salt treatment in broccoli was further confirmed by qRT-PCR. Consistent with the expression trend identified by comparative transcriptome sequence analysis, the expression level of *bolTLP1* was increased significantly in 4 h after exposure to salt stress (Figure 1C). In addition, the expression pattern of *bolTLP1* in the leaves of broccoli treated with 300 mM mannitol, which could mimic drought condition, was explored. Similar to observations under salt stress, the expression level of *bolTLP1* was significantly higher under drought stress than that observed under control condition (0 h) (Figure 1D).

### 2.3. Overexpression of bolTLP1 Increased Resistance to Salt and Drought Stresses in Arabidopsis

**Figure 2:**
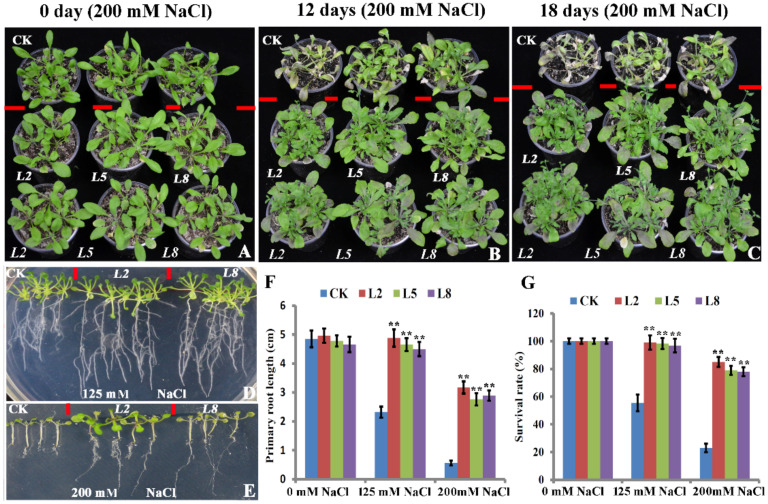
Phenotypes of transgenic *Arabidopsis* plants overexpressing *bolTLP1* under salt stress. (**A**–**C**): Phenotypes of 35S::*bolTLP1 Arabidopsis* at 0 day, 12 days and 18 days after irrigated with 200 mM NaCl in nutrient soil, respectively. (**D**,**E**): Phenotypes of 35S::*bolTLP1 Arabidopsis* seedlings at 15 days after growing in 1/2 MS medium with 125 mM and 200 mM NaCl, respectively. (**F**): Primary root length of 35S:*:bolTLP1 Arabidopsis* at 15 days after growing in 1/2 MS medium with 125 mM and 200 mM NaCl. (**G**): Survival rates of 35S::*bolTLP1 Arabidopsis* at 18 days after irrigated with 125 mM and 200 mM NaCl in nutrient soil. (Student’s *t*-test, ** *p* < 0.01; data are means ± SD (*n* ≥ 15)). The top row of (**A**–**C**) showed the vector controls (CK), and the middle and bottom rows showed the independent 35S::*bolTLP1 Arabidopsis* lines (*L2*, *L5* and *L8*).

To further uncover the roles of *bolTLP1*, a transformation vector containing the *bolTLP1* under the control of the enhanced CaMV 35S promoter was constructed, and used to transform *Arabidopsis*. A total of 26 independent 35S::*bolTLP1* transgenic lines were obtained (Figure S1). Under normal growth conditions, there were no phenotypic differences between the 35S::*bolTLP1* transgenic lines and the vector controls, thereby indicating that the overexpression of *bolTLP1* did not affect the growth and development of the transgenic lines in *Arabidopsis* (Figure 2A). To determine if overexpression of *bolTLP1* could improve resistance to salt and/or drought stress, 35S::*bolTLP1 Arabidopsis* lines were planted in soil, and the 25-day-old seedlings were subjected to 125 and 200 mM NaCl treatments, respectively. At 12 days after 200 mM NaCl treatment, the aerial organs of the vector controls were wilted, growth was retarded, and leaves were yellow and senescent. Compared with the vector controls, the 35S::*bolTLP1 Arabidopsis* was only slightly affected by the high salt condition (Figure 2B). At 18 days after salt stress, the growth of the remaining vector control plants was substantially inhibited. The survival rate of 35S::*bolTLP1 Arabidopsis* was significantly higher than that of the vector controls, and the aerial organs of the 35S::*bolTLP1 Arabidopsis* still showed normal growth. (Figure 2C,G). Under 125 mM NaCltreatment, although over 45% of the vector control plants died, almost all individual plants of the 35S::*bolTLP1 Arabidopsis* survived (Figure 2G). At both low (125 mM) and high (200 mM) NaCl treatments, the primary and lateral roots of the 35S::*bolTLP1 Arabidopsis* were stronger than those of the vector controls (Figure 2D–F).

**Figure 3:**
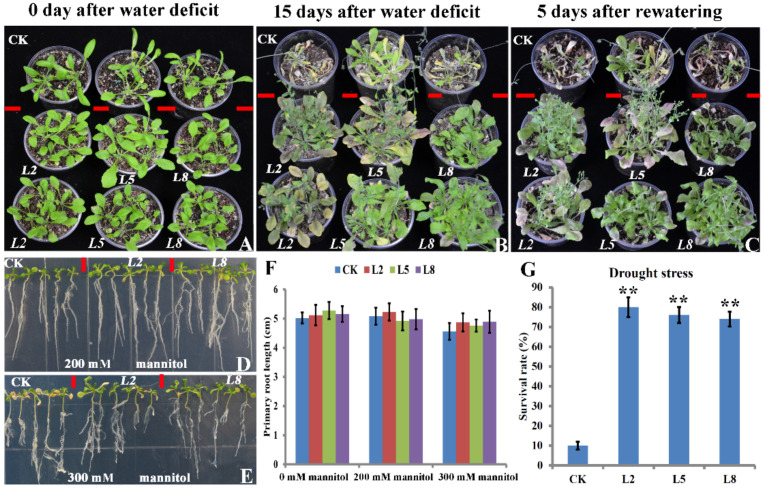
Phenotypes of transgenic *Arabidopsis* plants overexpressing *bolTLP1* under drought stress. (**A**,**B**): Phenotypes of 35S::*bolTLP1 Arabidopsis* at 0 day and 15 days after the water deficit. (**C**): Phenotypes of 35S::*bolTLP1 Arabidopsis* at 5 days after rewatering. (**D**,**E**): Phenotypes of 14-day-old 35S::*bolTLP1 Arabidopsis* seedlings growing in 1/2 MS medium with 200 mM and 300 mM mannitol, respectively. (**F**): Primary root length of 35S::*bolTLP1* transgenic *Arabidopsis* at 14 days after growing in 1/2 MS medium with 200 mM and 300 mM mannitol. (**G**): Survival rate of 35S::*bolTLP1 Arabidopsis* at 5 days after rewatering. (Student’s *t*-test, ** *p* < 0.01; data are means ± SD (*n* ≥ 20).) The top row of A, B and C showed the vector controls (CK), and the middle and bottom rows showed the independent 35S::*bolTLP1 Arabidopsis* lines (*L2*, *L5* and *L8*).

The 35S::*bolTLP1 Arabidopsis* plants were treated with water deficit. At 15 days after the water deficit, the leaves of the vector controls were seriously withered, and plant growth was inhibited. The growth and development of the 35S::*bolTLP1 Arabidopsis* were much better than those of the vector controls. In brief, the aerial organs of the 35S::*bolTLP1 Arabidopsis* were stronger than those of the vector controls, and their leaves were normal, although some of them also became senescent. The survival rate of 35S::*bolTLP1 Arabidopsis* was over 75%, whereas only 10% of the vector control plants survived after rewatering (Figure 3A–C,G). To further confirm the roles of *bolTLP1* under drought stress, the 35S::*bolTLP1 Arabidopsis* plants were treated with different concentrations of mannitol. At 200 mM mannitol, the aerial organs of the 35S::*bolTLP1 Arabidopsis* were larger than those of the vector controls. Similarly, at 300 mM mannitol, the growth and development of the aerial organs of the 35S::*bolTLP1 Arabidopsis* were still stronger than those of the vector controls (Figure 3D,E). Nevertheless, the roots, especially the primary roots, were not significantly affected by the mimic drought stresses (Figure 3F).

### 2.4. Transgenic Broccoli Overexpressing bolTLP1 Exhibited High Salt and Drought Tolerance

**Figure 4:**
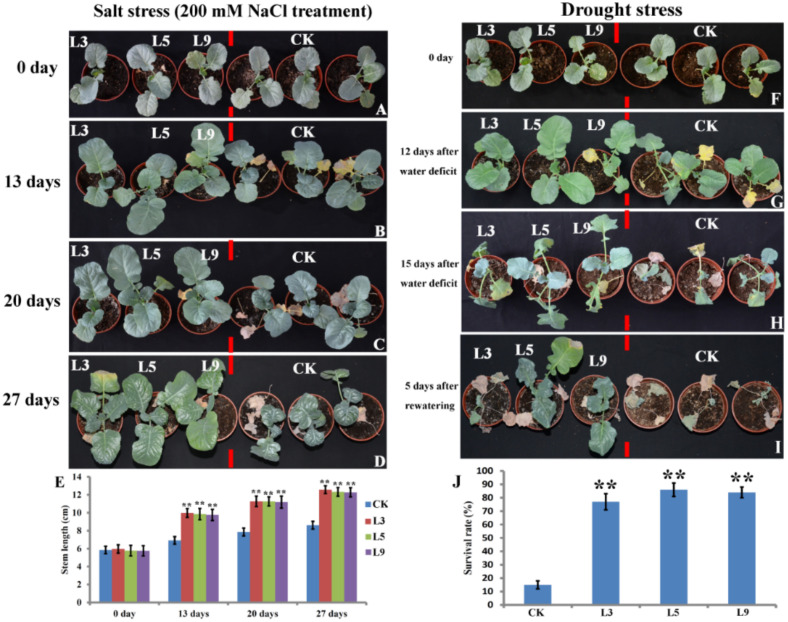
Phenotypes of transgenic broccoli plants overexpressing *bolTLP1* under salt and drought stresses. (**A**–**D**): Phenotypes of 35S::*bolTLP1* broccoli at 0 day, 13 days, 20 days and 27 days after irrigated with 200 mM NaCl, respectively. (**E**): Stem length of 35S::*bolTLP1* broccoli at 0 day, 13 days, 20 days and 27 days after irrigated with 200 mM NaCl. (Student’s *t*-test, ** *p* < 0.01; data are means ± SD (*n* ≥ 18)). (**F**–**H**): Phenotypes of 35S::*bolTLP1* broccoli at 0 day, 12 days and 15 days after water deficit, respectively. (**I**): Phenotypes of 35S::*bolTLP1* broccoli at 5 days after rewatering. (**J**): Survival rate of 35S::*bolTLP1* broccoli at 5 days after rewatering. (Student’s *t*-test, ** *p* < 0.01; data are means ± SD (*n* ≥ 15)). L3, L5 and L9 indicated the three independent 35S::*bolTLP1* broccoli lines. CK indicated the vector control plants.

*BolTLP1* was also overexpressed in broccoli. A total of 16 independent 35S::*bolTLP1* broccoli transgenic lines were obtained (Figure S1). Similar to the 35S::*bolTLP1 Arabidopsis*, the growth and development of the 35S::*bolTLP1* broccoli were unaffected under the normal growth conditions (Figure 4A). The 30-day-old 35S::*bolTLP1* broccoli seedlings were irrigated with 200 mM NaCl. At 13 days after the salt treatment, the growth of the 35S::*bolTLP1* broccoli was normal. Under these conditions, the vector controls grew slower and the first leaves showed signs of senescence (Figure 4B). As time went on, the 35S::*bolTLP1* broccoli showed slight salt damage, but their growth vigor was much better than that of the vector controls (Figure 4C–E). The growth and development of the 35S::*bolTLP1* broccoli under drought stress were also examined. The phenotypic data demonstrated that at 12 days after the water deficit, the aerial organs of the vector controls became withered, and their first and second leaves were senescing. Interestingly, at this same stage of water deficit, the growth of the 35S::*bolTLP1* broccoli was not considerably affected, and their aerial organs were larger and stronger than those of the vector controls. At 15 days after the water deficit, although the leaves of the 35S::*bolTLP1* broccoli and vector controls both showed withering, the growth vigor of the vector controls was inhibited to a greater degree than the 35S::*bolTLP1* broccoli. After rewatering, the majority of the 35S::*bolTLP1* broccoli (over 78%) survived, whereas almost all vector controls died (Figure 4F–J).

### 2.5. DEGs Were Confirmed between the 35S::bolTLP1 Broccoli and Vector Control

**Figure 5:**
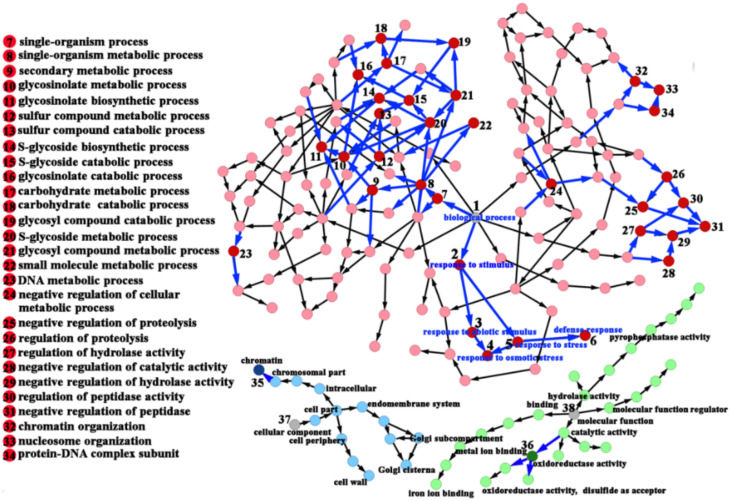
Regulatory network relationships of GO terms of the differentially expressed genes between 35S::*bolTLP1* broccoli and the vector control plants. Each node represents a GO term. GO terms in biological process, cellular component and molecular function were distinguished by red, blue and green color, respectively. The significantly enriched GO terms (corrected *p*-value < 0.01) are further marked by dark color, and their inclusion relations are highlighted by thick and blue arrows. The inclusion relations of other GO terms are marked by black arrows. Only those significantly enriched GO terms are serially numbered and their functional annotations were showed. The functional annotations of other enriched GO terms can be found in Table S2.

Comparative transcriptome analysis was conducted to identify the DEGs between the 35S::*bolTLP1* broccoli and the vector controls. In total, 34,258 genes showing transcriptional expression were detected, among which 3284 genes showed significantly differential expression levels (corrected *p*-value < 0.01). The expression levels of 2202 out of the 3284 DEGs were up-regulated in the 35S::*bolTLP1* broccoli, and the other approximately 33% of the DEGs displayed lower expression levels in the 35S::*bolTLP1* broccoli compared to the vector control plants (Table S1). GO functional annotations indicated that these DEGs were mapped to 933 GO terms in the biological process, 154 GO terms in the cellular component and 343 GO terms in the molecular function, among which 33, 1 and 1 GO terms were significantly enriched in biological process, molecular function and cellular component, respectively (corrected *p*-value < 0.01) (Figure 5, Table S2). The significantly enriched GO terms in the biological process were mainly involved in stress responses, glycosinolate and sulfur compound synthetic and metabolic process, and peptidase, proteolysis and hydrolase activity. The GO terms involved in oxidoreductase activity and chromatin organization were significantly enriched in the molecular function and the cellular components, respectively (Figure 5, Table S2).

### 2.6. Expression Profiles of Genes Involved in ABA, Ethylene and Auxin-Mediated Signaling Pathways Were Significantly Altered by Overexpression of bolTLP1

**Figure 6:**
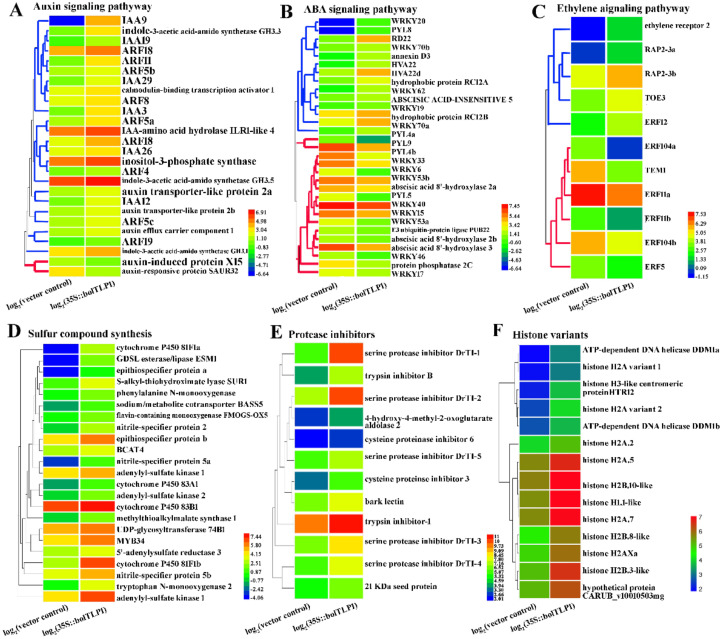
Transcriptional expression patterns of the differentially expressed genes involved in phytohormone-mediated signaling pathways (**A**–**C**), sulfur compound synthesis (**D**), protease inhibitors (**E**) and histone variants (**F**). The blue and red branches in (**A**–**C**) indicated the genes displayed significantly up-regulated and down-regulated expression in the 35S::*bolTLP1* broccoli compared with the vector control (corrected *p*-value < 0.01), respectively. Log_2_ (35S::bolTLP1) and log_2_ (vector control) indicated the log_2_FPKM of *bolTLP1* in 35S::*bolTLP1* broccoli and vector control plants, respectively, which represents the normalized expression level of *bolTLP1* detected by RNA-seq.

**Figure 7:**
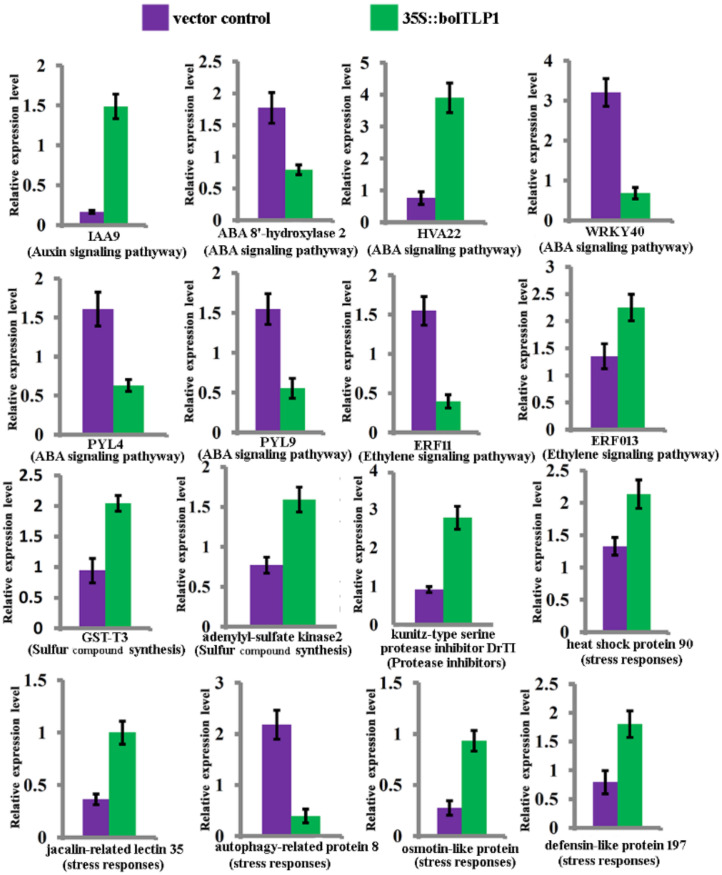
Identification of the relative expression levels of the representative genes by qRT-PCR. GST: glutathione S-transferase. PYL: abscisic acid receptor PYR/PYL/RCAR. ERF: ethylene-responsive transcription factor. IAA9: indol-yl-3-acetic acid 9. RD22: dehydration-responsive protein 22. 35S::bolTLP1: 35S::*bolTLP1* broccoli. Vector control: vector control plants.

According to the functional annotations of all DEGs by GO analysis, genes involved in the biotic and abiotic stress response processes were obviously predominant in the DEGs. At least 685 out of the 3284 DEGs (>21%) were functionally associated with stress response processes. Among the stress response-associated DEGs, approximately 70% of them displayed higher expression levels in the 35S::*bolTLP1* broccoli than those in the vector controls (Table S3). A large proportion of these DEGs were key regulators in phytohormone-mediated signaling pathways. In brief, twenty-six DEGs were involved in the auxin signaling pathway. Twenty-four out of the 26 DEGs displayed higher expression levels in the 35S::*bolTLP1* broccoli than those in the vector controls. Thirty-one genes involved in the ABA signaling pathway displayed differential expression patterns between the 35S::*bolTLP1* broccoli and the vector controls, among which 14 genes, such as *RD22*, *HVA22*, *RCI2A* and *ABSCISIC ACID-INSENSITIVE 5* (*ABI 5*), displayed significantly up-regulated expression patterns in the 35S::*bolTLP1* broccoli. At least 11 genes involved in the ethylene signaling pathway displayed differential expression patterns between the 35S::*bolTLP1* broccoli and the vector controls (Figure 6A–C). The expression levels of some of these DEGs were further verified by qRT-PCR (Figure 7).

### 2.7. Genes Involved in Sulfur Compound Synthesis and Hydrolase/Oxidoreductase Activity Were Significantly Inductively Expressed by the Overexpression of bolTLP1

Besides the DEGs involved in stress response, at least 62 DEGs were confirmed to map the GO terms associated with the glycosinolate and sulfur compound synthesis and metabolism, which displayed significant enrichment in the biological process. Forty-seven out of the 62 DEGs, such as genes encoding cytochrome P450 81F1, epithiospecifier protein, S-alkyl-thiohydroximate lyase, adenylyl-sulfate kinase, nitrile-specifier protein, flavin-containing monooxygenase, 5′-adenylylsulfate reductase and cytochrome P450 83B1, had significantly higher expression levels in the 35S::*bolTLP1* broccoli compared to the vector control plants. The majority of these genes play crucial roles in sulfur compound synthesis and metabolism (Figure 6D, Table S3).

The expression patterns of genes related to significantly enriched GO terms involved in the activity of diverse enzymes, such as the proteolysis, peptidase, hydrolase and oxidoreductase, were also analyzed. A total of 12 genes including genes encoding kunitz-type serine protease inhibitor, cysteine proteinase inhibitor, trypsin inhibitor and 4-hydroxy-4-methyl-2-oxoglutarate aldolase were associated with the activity of proteolysis and hydrolase, all of which exhibited significantly increased expression levels in the 35S::*bolTLP1* broccoli (Figure 6E). At least 192 DEGs were confirmed to be associated with the oxidoreductase activity. The majority of them (146 out of 192) also exhibited higher expression levels in the 35S::*bolTLP1* broccoli than those in the vector controls (Table S3). Moreover, 15 genes associated with the significantly enriched GO terms involved in chromatin organization, especially histone variants, were identified. All these genes showed increased expression levels in the 35S::*bolTLP1* broccoli (Figure 6F, Table S3).

### 2.8. Five Proteins Involved in Abiotic Stress Responses Were Confirmed to Interact with bolTLP1

**Figure 8:**
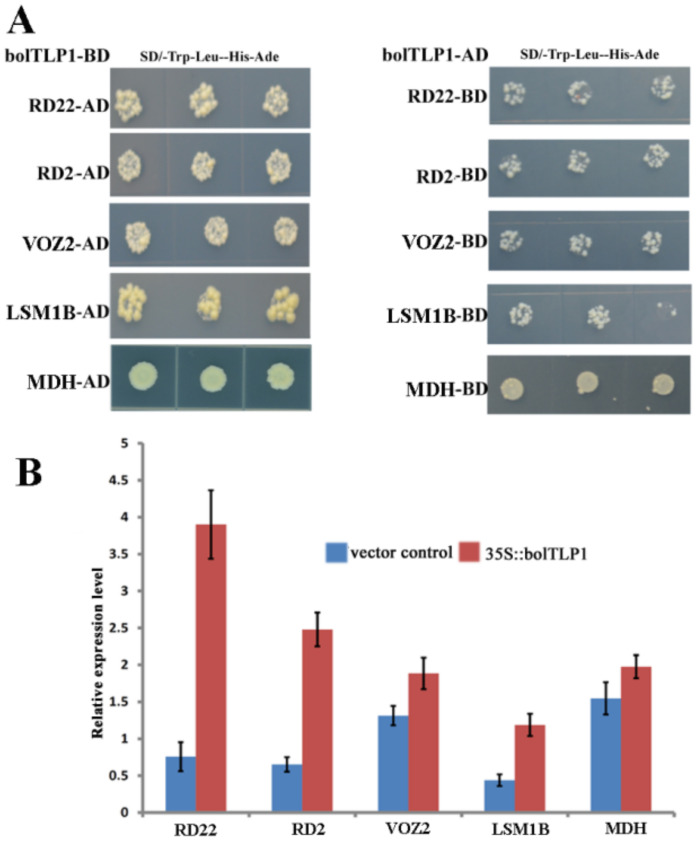
Identification and expression profiles of proteins interacting with bolTLP1. (**A**): Identification of proteins interacting with bolTLP1 by yeast two-hybrid assay. Three spots from left to right in each row indicated the 1:10 gradient dilution of yeast clone. (**B**): Relative expression levels of the five candidate genes, which encode proteins that interact with bolTLP1, in 35S::*bolTLP1* broccoli (35S::bolTLP1) and the vector control plants (vector control).

Yeast two-hybrid screening was conducted to identify proteins that could interact with bolTLP1. In total, 266 positive clones were identified and sequenced. These sequences were annotated as 20 diverse genes, among which *RD2*, *RD22*, *VOZ2*, *LSM1B* and *MDH* were functionally associated with stress responses in plants. The interaction of these five proteins with bolTLP1 was further confirmed by using bolTLP1 as the prey and RD2, RD22, VOZ2, LSM1B and MDH as the baits in the yeast two-hybrid assay (Figure 8A, Table S4). Quantitative expression analysis revealed that the expression levels of these stress response-associated genes were significantly higher in 35S::*bolTLP1* broccoli than those in the vector controls (Figure 8B).

## 3. Discussion

*TLPs* are present as a large group of PR-5 gene family in plants, and most *TLPs* were demonstrated to play a role in defending against pathogen [1,9,11,14,16]. Only a few *TLPs* have been demonstrated to function in response to abiotic stress. Nevertheless, in the present study, *bolTLP1*, a member of *TLP* subfamily, was first identified in broccoli. The expression profiles assay confirmed that *bolTLP1* displayed inducible expression patterns under salt and drought stresses. Overexpression of *bolTLP1* in broccoli, as well as in *Arabidopsis*, significantly increased the salt and drought tolerance of transgenic plants. These findings demonstrated that *bolTLP1* is an important positive response factor that plays a role in combatting salt and drought stress in broccoli, and suggested the potential applications of *bolTLP1* in breeding crops with high salt and drought tolerance via genetic engineering.

The molecular mechanisms by which the TLPs respond to abiotic stress remain largely unknown, although investigations have confirmed the roles of several *TLP* genes in cold temperature, salt and drought stresses [13,17,18,19]. In the present study, comparative transcriptome analysis revealed that the DEGs detected between the 35S::*bolTLP1* broccoli and the vector controls were mainly involved in the regulatory processes including “stress responses”, “glycosinolate and sulfur compound metabolic process”, “peptidase, proteolysis and hydrolase activity”, “oxidoreductase activity” and “chromatin organization” (Figure 5, Table S2). These results suggested that these regulatory processes should participate in *bolTLP1*-mediated tolerance to salt and drought stresses. Furthermore, the functional annotation analysis of the DEGs enriched in these regulatory processes confirmed that over 21% DEGs were involved in stress response processes, which represented the largest gene group in the DEGs. Remarkably, a large proportion of these DEGs were associated with phytohormone-mediated signaling pathways. For example, *RD2*, *RD22*, *ABI5*, *WRKY15*/*17*/*20*/*33*/*40*/*46*/*53*/*62*/*70*, *abscisic acid 8′-hydroxylase 2*, *annexin*, *PUB23*, *HVA22*, *RCI2A*, *RCI2B* and *PYL4*/*5*/*8*/*9* are the important members of ABA-mediated signaling pathway, which all displayed significantly differential expression levels between the 35S::*bolTLP1* broccoli and the vector controls (Figure 6B). Most of these genes were confirmed to play crucial roles in response to abiotic stresses in diverse plant species [34,35,36,37,38]. Interestingly, RD2 and RD22 were confirmed to directly interact with bolTLP1 (Figure 8). *RD2* and *RD22* are up-regulated by drought stress, salinity stress and exogenously supplied ABA. Their inducible expression has been used as a marker for abiotic stress [39,40]. In addition, at least 26 DEGs, such as auxin response factor (*ARF*) *4*/*5*/*8*/*11*/*14*/*18*/*19*, *auxin transporter 2*, *IAA3*/*9*/*12*/*19*/*26*/*29*, *inositol-3-phosphate synthase*, *indole-3-aceticacid-amido synthetase GH3*, *calmodulin-binding transcription activator 1* (*CAMTA 1*) and auxin-induced gene *X15* and *SAUR32* are involved in auxin-mediated regulatory processes (Figure 6A). Similarly, some of these genes such as *CAMTA 1* [41,42] and *inositol-3-phosphate synthase* [43,44] were identified to function in response to drought or salt stress. On the other hand, a few DEGs, such as *Ethylene receptor 2* and *ethylene responsive factor*s (*ERFs*) including *ERF04*, *ERF05*, *ERF09*, *ERF12*, *RAP2-3*, *RAP2-10*, *ERF11*, *ERF104*, *TEM1* and *TOE3*, are involved in ethylene signaling pathway (Figure 6C). Inducible expression of *RAP2-3*, as well as *RAP2-12* and *RAP2-2*, confers tolerance to anoxia, oxidative and osmotic stresses [45]. The *ERF6* overexpressing transgenic lines are hypersensitive to osmotic stress, while the growth of *erf5erf6* loss-of-function mutants is less affected by stress [46]. These results demonstrated that the overexpression of *bolTLP1* significantly affects the expression levels of a set of genes involved in the ABA, ethylene and auxin signaling pathways. Moreover. The homologues of a large proportion of these genes have been demonstrated to function in defending against abiotic stress in diverse plants. Our results also support the role of these genes in the *bolTLP1*-mediated tolerance to salt and drought stresses. This finding provided new insight into the roles of phytohormone-associated genes in response to abiotic stress in broccoli. Moreover, beside RD2 and RD22, three other proteins VOZ2, LSM1B and MDH were confirmed to directly interact with bolTLP1, and their genes displayed differently expressed pattens between the 35S::*bolTLP1* broccoli and vector controls (Figure 8). The investigations demonstrated that *VOZ* transcription factors act as positive regulators of salt tolerance in *Arabidopsis* [47]. *LSM1-7* are the components of the 5′–3′ mRNA decay pathway, which play a crucial role in regulating ABA signaling and modulating ABA-dependent expression of stress related transcription factors from the AP2/ERF/DREB family [48]. Overexpression of plastidic maize NADP-malate dehydrogenase (*ZmNADP*-*MDH*) increases tolerance to salt stress in *Arabidopsis* [49]. It suggested that these three abotic-stress associated genes could also play important roles in *bolTLP1*-mediated regulatory network.

Another remarkably overrepresented group of DEGs was associated with various enzymes, particularly proteolysis, hydrolase and oxidoreductase. The DEGs involved in proteolysis and hydrolase activity mainly function as lectin and kunitz-type protease inhibitors such as serine protease inhibitor, cysteine proteinase inhibitor and trypsin inhibitor (Figure 6E). Plant lectins and protease inhibitors constitute a class of proteins which play a crucial role in plant defense against insect and pathogen attacks [50,51]. Previous investigations confirmed that protease inhibitors also function in abiotic stress. The constitutive expression of a trypsin protease inhibitor confers tolerance to multiple stresses in transgenic tobacco [52]. *Oryzacystatin-I* (*OCI*) is a rice cysteine proteinase inhibitor. Ectopic overexpression of *OCI* can enhance drought stress tolerance in soybean and *Arabidopsis* [53]. *AtCYSa* and *AtCYSb* are two cysteine proteinase inhibitors from *Arabidopsis*. Overexpression of these inhibitors in transgenic yeast and *Arabidopsis* increases their resistance to high salt, drought, oxidative and cold stresses [54]. The present study confirmed that all these DEGs involved in proteolysis and hydrolase activity displayed significantly up-regulated expression in 35S::*bolTLP1* broccoli (Figure 6E). These findings indicated that the increased expression levels of these genes could also play important roles in promoting salt and drought tolerance of the *bolTLP1* transgenic plants.

A series of histone variants including *H1.1*, *H2A.1*, *H2A.2*, *H2A.5*, *H2B.3*, *H2A.7*, *H2B.8* and *DDM1* were also significantly overrepresented in the DEGs (Figure 6F). These genes are important epigenetic regulators, and display significantly higher expression levels in the 35S::*bolTLP1* broccoli than those in the vector controls (Figure 6F). *TaH2A.7* in wheat can enhance drought tolerance and promote stomatal closure when overexpressed in *Arabidopsis* [55]. In *Arabidopsis*, *DDM1* is an epigenetic link between salicylic acid metabolism and heterosis, in which salicylic acid can protect plants from pathogens and abiotic stress [56]. Consistently, the mutants of *DDM1* show higher sensitivity to NaCl stress than the wild type plants [57]. In addition, the data confirmed that over 60 genes involved in glycosinolate and sulfur compound synthetic and metabolic processes showed significant differential expression patterns in the *bolTLP1* overexpressing transgenic plants and the vector controls. Genes associated with sulfur compound synthesis were up-regulated expression in 35S::*bolTLP1* broccoli (Figure 6D). Sulfur compound such as glucosinolates are best known for their roles in plant defense against herbivores and pathogens as well as their cancer-preventive properties [58,59,60]. A few investigations confirmed that glucosinolates also contributed to abiotic stress [61,62,63,64]. These results suggested that the histone variants and these sulfur compound-associated genes may also play an important role in *bolTLP1*-mediated tolerance to abiotic stress.

## 4. Materials and Methods

### 4.1. Plant Materials and Stress Treatment

Homozygous broccoli seeds of KRJ-012 were kindly provided by Dr. Hanmin Jiang, Tianjin Kernel Vegetable Research Institute, Tianjin, China. The seeds were planted in a greenhouse with a 16 h/8 h light/dark cycle at 25 °C and 22 °C, respectively. The 25-day-old seedlings were subjected to salt and drought stresses. For the salt-stress treatment, at least 20 individual plants were irrigated with 200 mM NaCl. To mimic the drought stress, 30 individual plants were irrigated with 300 mM mannitol. The leaves of each plant were harvested at 0, 4, 8 and 24 h after these treatments, frozen immediately in liquid nitrogen, and then stored at −80 °C. To evaluate the salt tolerance of 35S::*bolTLP1* broccoli, at least 18 individual plants from three independent transgenic lines were continually irrigated with 200 mM NaCl (Tianjin Kemiou Chemical Reagent Co., Ltd., Tianjin, China), and three such replicated treatments were conducted. Similarly, a total of 48 individual plants from three independent 35S::*bolTLP1* broccoli lines were divided into three groups, and unirrigated to evaluate their drought tolerance. In addition, at least three 35S::*bolTLP1 Arabidopsis* lines in 1/2 MS medium or in soil were treated with 125 and 200 mM NaCl to mimic salt stress. Three 35S::*bolTLP1 Arabidopsis* lines in 1/2 MS medium were treated with 200 and 300 mM mannitol to mimic drought stress. The 35S::*bolTLP1 Arabidopsis* planted in soil were also unirrigated to further evaluate their drought tolerance. To validate the reliability of the assessments of salt and drought tolerance in 35S::*bolTLP1 Arabidopsis*, three replicates were performed for each treatment.

### 4.2. BolTLP1 Cloning and Phylogenetic Analysis

The ESTs of *bolTLP1* were identified from the broccoli transcriptome data, and the specific primers (*bolTLP1*-F/*bolTLP1*-R) were designed to amplify the full coding sequences of *bolTLP1* based on these expressed sequence tags (ESTs) (Table S5). Total RNAs were isolated from the 25-day-old broccoli seedlings by using TRIzol reagent (Invitrogen, Carlsbad, CA, USA) according to the manufacturer’s instructions. The first-strand cDNA was then synthesized using M-MLV reverse transcriptase (Promega, Madison, WI, USA), and the coding sequences of *bolTLP1* were amplified by PCR. Because the genome data of broccoli is still limited, to elucidate the sequence homology of *bolTLP1* with other *TLP* genes, those genes containing a TLP domain were identified from the genome database of *Brassica oleracea* var. *oleracea*, which is genetically closely related with broccoli (Table S6). The deduced amino acid sequences of *bolTLP1* and its homologous genes from *B. oleracea* var. *oleracea* were used to construct the phylogenetic tree using the neighbor-joining method by the MEGA 6.0 program with the following parameters: bootstrap value of 1000, poisson correction and pairwise deletion [65].

### 4.3. BolTLP1 Expression Profile Analysis

Total RNAs from the leaves of individual plants, which were harvested at different time points after imposing salt or drought stress, were isolated and reverse transcribed to cDNAs. Specific primers of *bolTLP1* (*qbolTLP1*-F/*qbolTLP1*-R) was designed by Primer premier 5.0 software (Table S5). The *bolActin* gene from broccoli was selected as the internal control and Faststart Universal SYBR Green Master (Roche, Basel, Switzerland) was used in quantitative real-time RT-PCR (qRT-PCR) assay. The relative expression levels of *bolTLP1* under salt or drought stress were calculated by the comparative 2^−ΔΔCT^ method. Three biological replicates and three technological replicates were performed to ensure the reliability of quantitative analysis.

### 4.4. Expression Vector Construction and Genetic Transformation

The full coding sequence of *bolTLP1* with incorporated *Nco* I and *Bst*E II restriction sites was amplified (Table S5) and cloned into the pEASY-T1 vector. The insert released from the pEASY-T1 vector with *Nco* I/*Bst*E II double digestion was sub-cloned into the pCAMBIA3301 binary vector. The recombinant 35S::*bolTLP1* plasmids and vectors without exogenous gene insertion (empty vectors) were transformed into *A. tumefaciens* strain LBA4404. *Agrobacterium*-mediated *Arabidopsis* genetic transformation was performed via the floral dip method. The transgenic *Arabidopsis* lines were screened by spraying 1:10,000 dilute Basta solution and further identified by PCR using specific primers (*bolTLP1*-F/*bolTLP1*-R) and combined primers (*35S*-F/*bolTLP1*-R). Simultaneously, the plants with empty vector were identified by PCR using specific primer pair (*35S*-F/*NOS*-R). The transgenic *Arabidopsis* plants were further verified by qRT-PCR using unique primers (*qbolTLP1*-F/*qbolTLP1*-R) and *atActin* as an internal control (Table S5). The 35S::*bolTLP1* plasmids and empty vectors were also transformed into broccoli using *Agrobacterium*-mediated method. In brief, broccoli seeds were rinsed with 75% ethanol for 2 min; 2% NaClO (Tianjin Fengchuan Chemical Reagent Co., Ltd., Tianjin, China) was then further used for surface sterilization of the seeds for 10 min. The sterilized seeds were planted on Murashige and Skoog (MS) medium with a 16 h/8 h light/dark cycle at 22 °C. The hypocotyls of 7-day-old broccoli seedlings were cut to 0.5 cm and pre-cultured on MS medium containing 1.5 mg/L 6-BA and 0.15 mg/L NAA for 2 days. Then, the truncated hypocotyls were dipped into a suspension of *Agrobacterium* containing the 35S::*bolTLP1* expression vector or an empty vector for 1 min. The infected explants were transferred onto co-cultivation medium with 1 mg/L 6-BA, 0.1 mg/L NAA and 100 μmol/L acetosyringone, and cultured for 2 days in the dark. Subsequently, the explants were washed with sterile water and transferred onto MS medium containing 1.6 mg/L 6-BA, 0.2 mg/L NAA and 200 mg/L cefotaxime for 10 days for callus induction. The differentiated explants were transferred into screening medium supplemented with 3 mg/L Basta for 2 weeks. Finally, buds that continued to differentiate were transferred to the 1/2 MS medium containing 1 mg/L IBA for root initiation. The transgenic broccoli plants were further verified by PCR using prime pair (*35S*-F/*bolTLP1*-R) and qRT-PCR using unique primers (*qbolTLP1*-F/*qbolTLP1*-R) (Table S5).

### 4.5. Transcriptome Sequencing and Data Analysis

Leaves from six 30-day-old individual plants of per independent 35S::*bolTLP1* broccoli line were equally mixed and used to perform three batches of independent RNA isolation. Equal amounts of RNA from each independent 35S::*bolTLP1* broccoli line were then used to construct sequencing library. In total, two such sequencing libraries from two independent 35S::*bolTLP1* broccoli lines and one sequencing library from the vector controls were constructed. The sequencing reaction was conducted by the Illumina HiSeq^TM^ 2500 sequencing platform (Beijing Genomics Institute, Shenzhen, China) with three technological replicates. The clean reads were annotated and mapped to the reference genome of *B. oleracea* var. *oleracea*. The expression levels of genes were calculated by the fragments per kilobase of transcript sequence per million base pairs sequenced (FPKM). The significantly expression levels of genes between the 35S::*bolTLP1* broccoli and the vector controls were identified based on the thresholds: |log_2_ (fold-change (35S::*bolTLP1* broccoli/vector control))| > 1 and corrected *p*-value < 0.05. Gene Ontology (GO) analysis of the differentially expressed genes (DEGs) was performed by the agriGO platform. Available online: http://bioinfo.cau.edu.cn/agriGO/ (accessed on 4 May 2018).

### 4.6. Differentially Expressed Gene Identification by qRT-PCR

The gene expression profiles detected by comparative transcriptome analysis were verified by qRT-PCR by using the specific primer pairs (Table S5). Three independent 35S::*bolTLP1* broccoli lines (Line 3, Line 5 and Line 9) were used to perform three biological replicates. Similar to RNA isolation in transcriptome sequencing, RNAs from leaves of six randomly selected 30-day-old plants per 35S::*bolTLP1* broccoli line were isolated and reverse transcribed to cDNAs. The comparative 2^−ΔΔCT^ method was conducted to calculate the relative expression levels of genes using *bolActin* as an internal control. To further ensure the reliability of qRT-PCR, three technological replicates were carried out.

### 4.7. Yeast Two-Hybrid Screening and Assays

The proteins which interact with bolTLP1 were screened using Matchmaker^TM^ Gold Yeast Two-Hybrid System (Clontech, Mountain View, CA, USA) according to the manufacturer’s instructions. In brief, the full-length coding sequences of *bolTLP1* with the *Nco* I and *Bam*H I restriction enzyme sites were constructed into the bait vector pGBKT7 (Table S5). The recombinant bait vector pGBKT7-*bolTLP1* was transformed into the Y2H Gold yeast train, and the autoactivation and toxicity of bolTLP1 were detected. The positive pGBKT7-*bolTLP1* yeast then was mixed and mated with the universal *Arabidopsis* Mate & Plate library (Clontech, Mountain View, CA, USA). The positive mating yeast was screened on SD/-Trp-Leu-His-Ade medium with 0.5 mM 3-Amino-1, 2, 4-triazole (3-AT), which could effectively inhibit the autoactivation of bolTLP1. The plasmids from the mating yeast were isolated and used as a template to amplify the candidate genes using the universal primers (T7-F/3′AD-R) (Table S5). To further confirm the relationship of bolTLP1 and its candidate interacting proteins, the broccoli homologs of the positive *Arabidopsis* genes were cloned and inserted into the bait vector pGBKT7. In this case, bolTLP1 was inserted into the prey vector pGADT7 (Table S5). Subsequently, the yeast two-hybrid experiments were conducted according to the manufacturer’s instructions. Transcript expression levels of the broccoli genes encoding proteins interacting with bolTLP1 were analyzed by qRT-PCR as mentioned above (Table S5).

## 5. Conclusions

**Figure 9:**
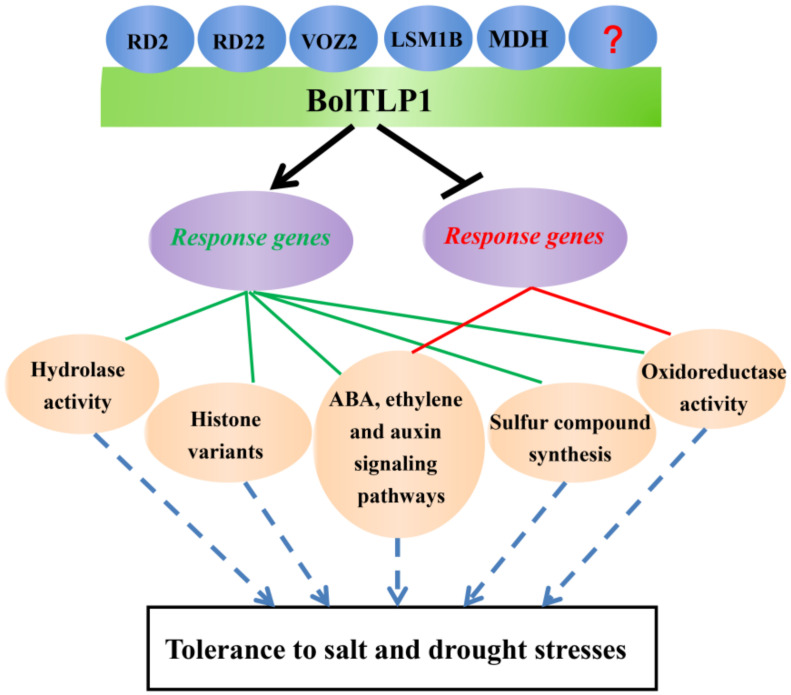
A proposed model indicating the regulatory processes of *bolTLP1* in increasing the tolerance to salt and drought stresses in broccoli. This model indicated that in response to salt and drought stresses, bolTLP1 may directly interact with RD2, RD22, VOZ2, LSM1B and MDH to positively regulate a series of genes involved in phytohormone (ABA, ethylene and auxin)-mediated signaling pathways, hydrolase activity, oxidoreductase activity, sulfur compound synthesis and histone variants. Meanwhile, the expression of several genes involved in phytohormone (ABA, ethylene and auxin)-mediated signaling pathways and oxidoreductase activity were inhibited by the overexpression of *bolTLP1*. The green font and lines showed the positive regulation of response genes. The red font and lines showed the negative regulation of response genes.

Overexpression of *bolTLP1* significantly increased the salt and drought tolerance in both *Arabidopsis* and broccoli. BolTLP1 may directly interact with stress response-associated proteins RD2, RD22, VOZ2, LSM1B and MDH to regulate a series of genes involved in phytohormone (ABA, ethylene and auxin)-mediated signaling pathways, hydrolase/oxidoreductase activity, sulfur compound synthesis, and histone variants, which could play important roles in *bolTLP1*-mediated tolerance to salt and drought stresses in broccoli (Figure 9). *BolTLP1* is a potential candidate gene in breeding crops with high tolerance to abiotic stress via genetic engineering.

## Data Availability

Data are contained in Supplementary Materials.

## Supplementary Materials

The following are available online at https://www.mdpi.com/article/10.3390/ijms222011132/s1.
