# Supplementary material for: BolTLP1, a Thaumatin-like Protein Gene, Confers Tolerance to Salt and Drought Stresses in Broccoli (Brassica oleracea L. var. Italica)
Source: Int J Mol Sci. 2021 Oct 15;22(20):11132. doi: 10.3390/ijms222011132 (PMC8537552; doi:10.3390/ijms222011132)
Supplement: Supplementary file 1 [file ijms-22-11132-s001.zip › Supplementary files/Supplementary Fig. S1.pdf]

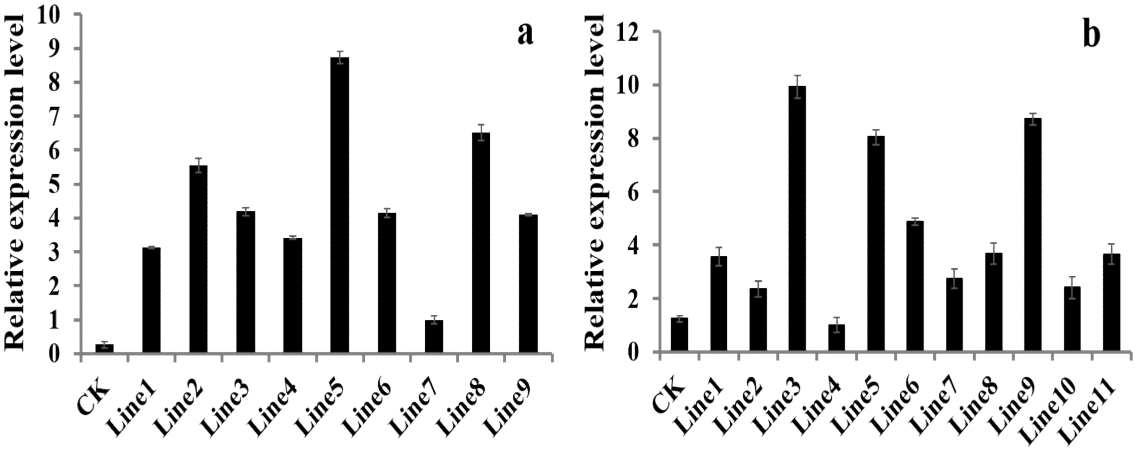

Supplementary Figure S1 Expression levels of *bolTLP1* in *bolTLP1* overexpressing transgenic *Arabidopsis* (a) and broccoli (b). CK in (a) and (b) indicated the vector control plants. Line1-9 in (a) indicated the representative independent *35S::bolTLP1* transgenic *Arabidopsis* lines. Line1-11 in (b) indicated the representative independent *35S::bolTLP1* transgenic broccoli lines.
