# Supplementary material for: BolTLP1, a Thaumatin-like Protein Gene, Confers Tolerance to Salt and Drought Stresses in Broccoli (Brassica oleracea L. var. Italica)
Source: Int J Mol Sci. 2021 Oct 15;22(20):11132. doi: 10.3390/ijms222011132 (PMC8537552; doi:10.3390/ijms222011132)
Supplement: Supplementary file 1 [file ijms-22-11132-s001.zip › Supplementary files/Supplementary Table S4.pdf]

Supplementary Table S4. Proteins interacting with bolTLP screened by yeast two-hybrid assay.

**Accession Number**	**Gene Symbol**	**Functional Annotation**	**No. of Clones**
AT2G21620	RD2	Response to desiccation	68
AT5G25610	RD22	Response to desiccation	31
AT3G14080	LSM1B	Small nuclear ribonucleoprotein family protein	22
AT3G47520	MDH	Protein with NAD-dependent malate dehydrogenase activity	19
AT1G43170	RP1	Ribosomal protein 1	16
AT2G42400	VOZ2	VOZ transcription factor which acts as positive regulator of several salt-responsive genes	16
AT3G27810	MYB21	R2R3-MYB transcription factor gene family	13
AT1G72010	Unreported	TCP family transcription factor	12
AT3G57790	Pectin lyase-like 1	Pectin lyase-like superfamily protein	11
AT1G02410	COX11	Cytochrome c oxidase 11 protein family	11
AT5G11790	NDL2	N-MYC downregulated-like 2	10
AT3G01015	TPX2	TPX2 protein family	9
AT2G28590	Protein kinase 5b	Protein kinase superfamily protein	9
AT5G22790	RER1	Reticulata-related 1	7
AT3G13800	Unknown	Metallo-hydrolase/oxidoreductase	4
AT5G16760	ITPK1	Inositol 1,3,4-trisphosphate 5/6-kinase	2
AT1G26650	Unreported	Son of sevenless protein	2
AT2G35240	MORF6	Member of MORF family	2
AT4G17510	UCH3	Ubiquitin C-terminal hydrolase 3	1
AT5G01010	Unreported	Retinal-binding protein	1
Total			266
