# Supplementary material for: BolTLP1, a Thaumatin-like Protein Gene, Confers Tolerance to Salt and Drought Stresses in Broccoli (Brassica oleracea L. var. Italica)
Source: Int J Mol Sci. 2021 Oct 15;22(20):11132. doi: 10.3390/ijms222011132 (PMC8537552; doi:10.3390/ijms222011132)
Supplement: Supplementary file 1 [file ijms-22-11132-s001.zip › Supplementary files/Supplementary Table S5.pdf]

Supplementary Table S5. Primers used in the study.

Primes		Sequences (5'-3')
BolTLP1 (full coding sequences)		bolTLP1-F:ATGATTTATCAAAAAACACTTCTCACA
		bolTLP1-R:TCACGGACAGAAAATGACATTGTAG
BolTLP1 with restriction enzyme sites (for construction of expression vector)		Nco I-bolTLP1-F:GCCCATGGCTTGATTAGTTTC
		Bst EII-bolTLP1-R:GGTCACCGATAATCACACTTCC
BolTLP1 with restriction enzyme sites (for construction of bait vector)		BD-bolTLP1-F:CCATGGATGATTTATCAAAAAACAC
		BD-bolTLP1-R:GGATCCTCACGGACAGAAAATG
BolTLP1 with restriction enzyme sites (for construction of prey vector)		AD-bolTLP1-F:GGATCCATGATTTATCAAAAAACAC
		AD-bolTLP1-R:GAGCTCTCACGGACAGAAAATG
Primes used in construction of bait vector	*LSM1B*	F:CATATGATGTCGTGGGCTGGTCCT
		R:GTCGACTTAATCGAAGTCAAGAAACTCCATT
	RD2	F:CATATGATGTCTCTAACCCTAAAAATCTC
		R: GTCGACCTACTGGACGCCG
	VOZ2	F: CATATGATGGGATGCTTCGG
		R: GTCGACTTAATTTGCTTGTGCTG
	RD22	F:CATATGATGGCGGCTTGTCTTCCAT
		R:GTCGACCTAGTAGCTGAACCAAACAACATGG
	MDH	F:CATATGATGGCAACAGCAACATCAGC
		R:GTCGACCTAGTTAGCTGCTGCAGCAGCT
Universal primers	*T7*	F: TAATACGACTCACTATAGGGCCA
	3'BD	R: AAAAGCAAAATTTTGGATTCTCA
	3'AD	R: CACTTGAACGCCCCAAAAAGTCATAG
	35S	F: AACAGAACTCGCCGTAAAG
	NOS	R: TTGCGGGACTCTAATCATAAAAC

	Genes	sequences (5'-3')
Primes used in qRT-PCR	*bolTLP1*	*qbolTLP1*-F:CGGTGTTGAAGACGGAGGAA
		qbolTLP1-R:CCAGTCTCTTGATAATCACAC
	jacalin-related lectin 35	106295110-F:TGGCGAAAAAGTTGGATGCTCA
		106295110-R:CACGGATGTGATGTAATCTTCTGGA
	PYL9	106298389-F:CCCCTGTTCATCTCGTTTGGTCT
		106298389-R:ATCACCTTTCATCACACACCTGCTG
	ERF13	106298736-F:CCACTTCCTCATCGTCACTGTCTCA
		106298736-R:TTCTTGTCTTTTGATTTGGTGCCCT
	ERF11	106301710-F:CGGAATCAAAGAAACCCGTTACAG
		106301710-R:GCTCTCGTTGTTGTCGACCATGTA
	adenylyl-sulfate kinase 2	106302172-F:AGCGACTGCCTCTAAGTGACGGTT
		106302172-R:CGGAGATCGGATGTAAAGAAGCAG
	IAA9	106313751-F:AGATGACAAAGCCACTATCAGCCTC
		106313751-R:TGGTAGGAAGCAAAGGAAAGAAGG
	HVA22-like	106333342-F:CTGTGGTGATGCTGCTTTAT
		106333342-R:TGTTCCCTCACGACTCTGTT
	DrTI	106319958-F:GAAGAGGTGAAGGACTACAACGGAA
		106319958-R:TGATGCCAAGTGGACAAAAAAAGTA
	glutathione S-transferase T3	106319962-F:GAGTACTGAACAAAAAGGTGGTGCC
		106319962-R:ACCAAATCATTGATCCTAGCCCAT
	heat shock protein 90	106322283-F:GTGAGAGCAAGAAGGCTGTGGA
		106322283-R:GGACTCTTCCAGTTTCAGACCCTC
	WRKY 40	106327894-F:ATGCGAGTTGAAGAAGATTCCACG
		106327894-R:AGGACTGACTTGGTCTCTCTCGGTA
	autophagy-related protein 8e	106335452-F:AAGCGTGACAACGATTACGAAAAGA
		106335452-R:TCTCAGCCTTTTCCACAATCACC
	ABA 8'-hydroxylase 2	106336684-F:TTGCTAGTGGTGTTTCTTCAATGGC
		106336684-R:TAGGGTTCTCTGTGTAGAGGCGGA
	osmotin-like protein	106337582-F:TGCTCCCACTACAACGGAAAATTC
		10.997606337582-R:CGAGAGAGACACCGTAGGAGGATAA
	defensin-like protein 197	106339924-F:TAGCATTGTTCATCTTCTTCCTCGC
		106339924-R:GCCTTTAGCACCTTTGAAACTCCTG
	PYL4	106342302-F:TAGGAGCCGACGAGAAGTTCAAAT
		106342302-R:TTCTTCTCGCATTTACCGCCTTT
	LSM1B	103870068-F: CTGGTCCTGAAGATATTTACC
		103870068-R: TCCACGGATTACATAGAGGC
	RD2	*RD2*-F:GGTCCCAACAGCAAACACGC
		RD2-R:CAGCCGCAGGCTTCAATCTC
	RD22	103837093-F:GTCTCCGCCATCTCTTTACGCC

		103837093-R:TGTTCCCTTACCCTTGCCAGTGTC
	MDH	114620-F: CCGTAACGAAAGCACAAACA
		114620-R: AAGGACCAGTGAAATCACGAAC
	VOZ2	106336717-F:GTTCCCGACGAGTCTGAGTT
		106336717-R:CTAATCCCGCCTGGTCTTAA
	Actin	*BolActin*-F: GCTCCTCTTAACCCAAAGGC
		BolActin-R: CACACCATCACCAGAATCCAGC
		AtActin-F: GGTAACATTGTGCTCAGTGGTGG
		AtActin-R: AACGACCTTAATCTTCATGCTGC

Notice: The underlines indicated the restriction enzyme sites. CK indicated the vector controls. OE indicated bolTLP1 overexpressing transgenic lines.
